# PD124 Genicular Artery Embolization For The Treatment Of Knee Osteoarthritis: A Systematic Review And Meta-analysis

**DOI:** 10.1017/S0266462324003611

**Published:** 2025-01-07

**Authors:** Yadira González-Hernández, Aránzazu Hernández-Yumar, Aythami De Armas-Castellano, Cristina Valcárcel-Nazco, Montse Carmona-Rodríguez, Mar Trujillo-Martín, Tasmania del Pino-Sedeño

## Abstract

**Introduction:**

The genicular artery embolization (GAE) procedure has been recently adopted for the management of pain secondary to inflammatory diseases of the locomotor apparatus. The number of studies assessing its use in patients with knee osteoarthritis (KO) has been increasing in recent years.

**Methods:**

We included two randomized controlled trials (RCTs) evaluating the use of GAE in patients with chronic pain secondary to KO. A cost analysis was also conducted to compare the costs of GAE and standard treatment from the perspective of the Spanish National Health System over a time horizon of one year. The potential improvement in quality-adjusted life-years necessary to consider GEA as cost effective for this indication was estimated. We also ran extensive sensitivity analyses.

**Results:**

Estimates for pain showed contradictory results, and no significant differences were observed between the two treatments with respect to overall function, health-related quality of life (HRQoL), and need for pain medication. No serious complications or major adverse events were observed. The quality of evidence was assessed by GRADE as moderate to low. The cost analysis showed that GAE results in an incremental cost of EUR3,432.37 per patient. Sensitivity analyses revealed a wide range within which the incremental cost can vary.

**Conclusions:**

There are insufficient data to discern any differences between GAE and standard treatment for patients with KO in terms of pain, function, HRQoL, need for analgesics, and rates of adverse events and complications. Larger RCTs are required to evaluate the effect of GAE in patients with chronic pain secondary to KO and to determine whether its additional cost is warranted.

